# Construction and validation of a gene signature related to bladder urothelial carcinoma based on immune gene analysis

**DOI:** 10.1186/s12885-022-09794-9

**Published:** 2022-08-27

**Authors:** Peng Xing, Zhengming Jiang, Yang Liu

**Affiliations:** Department of Urology, The First Hospital of China Medical University, Shenyang, 110013 P.R. China

**Keywords:** BLCA, ssGSEA, Gene signature, Immunotherapy, TCGA

## Abstract

**Background:**

This study developed a gene signature associated with a malignant and common tumor of the urinary system, the Bladder Urothelial Carcinoma (BLCA).

**Methods:**

The Cancer Genome Atlas (TCGA) database was searched to obtain 414 BLCA samples and the expression spectra of 19 normal samples. Single-sample Gene Set Enrichment Analysis (ssGSEA) was conducted to determine the enrichment levels in the BLCA samples of the 29 immune genes. Unsupervised hierarchical clustering, gene set enrichment analysis (GSEA), single-factor Cox analysis, least absolute shrinkage and selection operator (LASSO) regression models, and GEO queues were used to determine the BLCA immune gene subtype, analyze the biological pathway differences between immune gene subtypes, determine the characteristic genes of BLCA associated with prognosis, identify the BLCA-related genes, and verify the gene signature, respectively.

**Results:**

We identified two immune gene subtypes (immunity_L and immunity_H). The latter was significantly related to receptors, JAK STAT signaling pathways, leukocyte interleukin 6 generation, and cell membrane signal receptor complexes. Four characteristic genes (*RBP1*, *OAS1*, *LRP1*, and *AGER*) were identified and constituted the gene signature. Significant survival advantages, higher mutation frequency, and superior immunotherapy were observed in the low-risk group patients. The gene signature had good predictive ability. The results of the validation group were consistent with TCGA queue results.

**Conclusions:**

We constructed a 4-gene signature that helps monitor BLCA occurrence and prognosis, providing an important basis for developing personalized BLCA immunotherapy.

## Background

Bladder cancer is amongst the ten most common forms of cancers worldwide. In 2020, approximately 213,000 deaths from approximately 573,000 new cases of bladder cancer were estimated to have occurred [1]. The incidence of bladder cancer in men is approximately three to four times that in women, but most women are already in an advanced stage of the disease when they are diagnosed with bladder cancer, thus leading to a worse prognosis. There are several subtypes of bladder cancer, including bladder urothelial carcinoma (BLCA), squamous cell carcinoma, adenocarcinoma, small cell carcinoma, and sarcoma, among which the first is the main one [2]. There has been a rising trend in the incidence and mortality rates of BLCA over the years. With the aging of the population and the increase in environmental pollution, the public health burden of BLCA will also increase [3, 4]. The usual procedures used for the treatment of BLCA are transurethral resection, radical cystectomy, radiotherapy, and chemotherapy. However, BLCA is aggressive and highly recurrent and has a poor prognosis. Therefore, the diagnosis of BLCA, its treatment, and the five-year survival rates have remained basically unchanged [2, 5–7]. Traditional therapies can no longer meet the current status of BLCA treatment. In recent years, with the development of tumor immunology and the introduction of immunosuppressive agents in cancer treatment, the treatment of bladder cancer is expected to progress [8]. A large number of studies has analyzed the immune characteristics of BLCA patients from the perspective of immune infiltration [9–11]. However, there are still few studies on exploring new potential prognostic and immunotherapeutic biomarkers of BLCA with respect to tumor immune interaction.

The number of studies on the role played by the tumor immune microenvironment in tumor development and tumorigenesis has been increasing with the rapid advancement of the next-generation sequencing technology [12, 13]. The tumor microenvironment has a huge impact on the occurrence and development of BLCA and also helps to predict the response of patients with BLCA to immunotherapy [14, 15]. Immunotherapies such as immune checkpoint blockade, cytokine therapy, and cell therapy for the treatment of cancer have evolved in recent years [16, 17]. Immunotherapy is a potential neoadjuvant therapy for the treatment of BLCA. However, only a few patients benefit from this treatment [18–20]. The increase in immune cell infiltration in BLCA is an effective indicator of the response to immunotherapy [21, 22]. Significant correlation between immunotherapy and Human Leukocyte Antigen (HLA) and PD-L1 expression was observed [23–25]. The accumulation of somatic mutations can promote the occurrence and development of tumors and is conducive to the expression of neoantigens [26]. Previous cumulative studies have also shown that the tumor mutational burden (TMB) can predict the prognosis of patients with tumors [27, 28], and recent studies have begun to explore the correlation between the TMB and immunotherapy response [29, 30]. Therefore, it is necessary to understand the key factors affecting immunotherapy in BLCA.

In this study, the abundance level of immune cells in BLCA were quantified applying single-sample Gene Set Enrichment Analysis (ssGSEA). We further determined the immune subtype of BLCA according to the ssGSEA score. The differential genes between the BLCA immune subtypes were determined, and the intersection with the immune gene set was used to obtain BLCA-related immune genes related to the prognosis. Next, a new gene signature was constructed based on these immune genes related to the prognosis of BLCA to calculate the survival risk of the patient. In addition, according to the clinicopathological parameters and the signature, a nomogram to predict the 1-, 3-, and 5-year overall survival rates was constructed. Our research provides a new potential gene signature and immunotherapy classification method for the treatment of BLCA to promote the development of a personalized and precise treatment approach.

## Materials and methods

### Downloading and processing the research data

We downloaded the research data of this study (TCGA-BLCA) from The Cancer Genome Atlas (TCGA) database, including the transcriptome data and clinical information associated with 414 BLCA samples and 19 normal samples. We used "bladder cancer” (title) and "series" (entry type) and "Homo sapiens" (organism) as keywords in the GEO database ( http://www.ncbi.nlm.nih.gov/geo/) to obtain two datasets (GSE32894 and GSE31684) and series matrix files of related microarray platforms. Then, the two datasets were merged, and the expression data of that dataset were batch standardized using the "sva" package in R, so as to obtain a normalized gene expression matrix file for external validation analysis. The TCGA database provided the mutation data for the BLCA samples. In addition, the 2,483 immune genes were obtained from the Immunology Database and Analysis Portal (ImmPort (https://www.immport.org/). The imvigor210 immunotherapy dataset was taken from a study published by Sanjeev Mariathasan et al. [31].

### Cluster analysis of BLCA samples

To obtain the ssGSEA score subsequent to quantifying the level of enrichment of the immune cells in every BLCA sample, single-sample gene enrichment analysis (ssGSEA) was applied in the TCGA-BLCA cohort. Next, to determine the immune subtype of BLCA, an unsupervised cluster analysis of BLCA afflicted patients was conducted based on the ssGSEA score.

### Analysis of the correlation between BLCA immune genome subtypes and molecular characteristics

ESTIMATE is an algorithm used to estimate the immune scores, matrix scores, and ESTIMATE scores in tumor microenvironments. Using the ESTIMATE algorithm, the tumor purity, matrix content, and level of immune cell infiltration of each BLCA sample were calculated. To establish the relationship between the different BLCA immune infiltration and immune subtypes, the stromal, immune, and ESTIMATE scores of the various immune subtypes were compared. The differences in HLA genes in the BLCA immune subtypes were assessed, and their levels of expression in each subgroup determined. In addition, for comparing the ratio of immune cell subgroups between the BLCA immune subtypes, and for evaluating tumor-infiltrating immune cells in the BLCA samples, the CIBERSORT deconvolution algorithm was used [32]. The CIBERSORT platform (https://cibersortx.stanford.edu/) provided the gene expression characteristic matrix of the 22 tumor-infiltrating immune cells.

### Functional analysis between different immune subgroups and risk groups

Gene enrichment analysis (GSEA) was used to clarify differences in molecular mechanisms between different immune gene subtypes and between different risk groups. Gene Ontology (GO) analysis was based on c5.go.v7.symbols.gmt and consisted of three parts: biological process (BP), molecular function (MF), and cellular component (CC). Kyoto Encyclopedia of Genes and Genomes (KEGG) analysis was performed using c2.cp.kegg.v7.symbols.gmt [33–35]. Statistically significant values were considered to have an FDR < 0.25 and a *P* < 0.05.

### Identification of differentially expressed genes (DEGs) between different immune subgroups

The ‘limma’ package in R was used for screening the DEGs amongst the various immune subtypes, with a False Discovery Rate (FDR) < 0.05 criterion.

### Identification of the significance of immune genes in BLCA prognosis

We used the intersection between DEGs and immune gene sets to identify immune genes related to BLCA. Using a *P* < 0.001 as the screening criterion, Univariate Cox Regression Analysis was applied.

### Construction and verification of the BLCA gene signature

We used the least absolute shrinkage and selection operator (LASSO) to analyze and construct the BLCA-related gene signature. The LASSO algorithm reduces the number of variables by constructing a penalty function to effectively avoid overfitting. The penalty parameters of the model are based on the minimum partial likelihood deviation λ. This value was determined by tenfold cross-validation. The characteristic genes and corresponding coefficients contained in the risk signature were determined by the λ-value. The formula of the BLCA gene signature was as follows: BLCA risk score = gene1 × coef1 + gene2 × coef2 + gene3 × coef3 + …… + gene(n) × coef(n), with the regression coefficient being represented as ‘coef’. The BLCA samples from the TCGA cohort were segregated into high-risk and low-risk categories based on the median values of the risk scores. To evaluate the BLCA gene signature value and the prognostic ability, multivariate and univariate Cox regression analyses, survival curve, and Receiver Operating Characteristic (ROC) curve were applied. In addition, we used a test cohort and GEO queue as verification queues, and verified the stability and reliability of the gene signature using the same method.

### Analyzing the correlation between the built gene signature, the immune microenvironment, clinical characteristics, and the immune therapy response

A heatmap was used to analyze the correlation between the gene signature and the immune cell genes. We also explored the correlation between BLCA-related genes and clinical characteristics, including age, sex, grading, and stage. The TCGA database provided the clinical feature information. The immunophenoscore (IPS) is a good predictor of CTLA-4 and PD-1 reactivity, and immune checkpoint inhibitor immunoformidal scoring files were obtained from the Cancer Immunogenic Group database [36–38]. Based on the IPS files, we analyzed and predicted the differences in the responses in the group to immunotherapy using CTLA-4 and PD-1 blockers. Moreover, we also predicted the survival difference between high-risk and low-risk groups based on imvigor210.

### Development and verification of the nomogram

Based on the risk score and the independent clinicopathological characteristics, a nomogram was developed for evaluating the survival probability for the 1-, 3-, and 5-year periods of each BLCA patient. A calibration chart and a time-dependent ROC curve were used to verify the nomogram performance.

### Statistical analysis

R v4.0.3 (R Foundation for Statistical Computing, Vienna, Austria) was used to conduct all statistical analyses. The “SurvivalROC” package was used to calculate the area below the ROC curve (AUC). An AUC ≥ 0.70 and an AUC ≥ 0.6 were defined as significant and acceptable predictors, respectively. The Kaplan–Meier method and order-to-peer test were used for survival analysis. Statistical significance was considered in case of *P*-value < 0.05.

## Results

### Identification of the BLCA immune gene subgroup

**Fig. 1:**
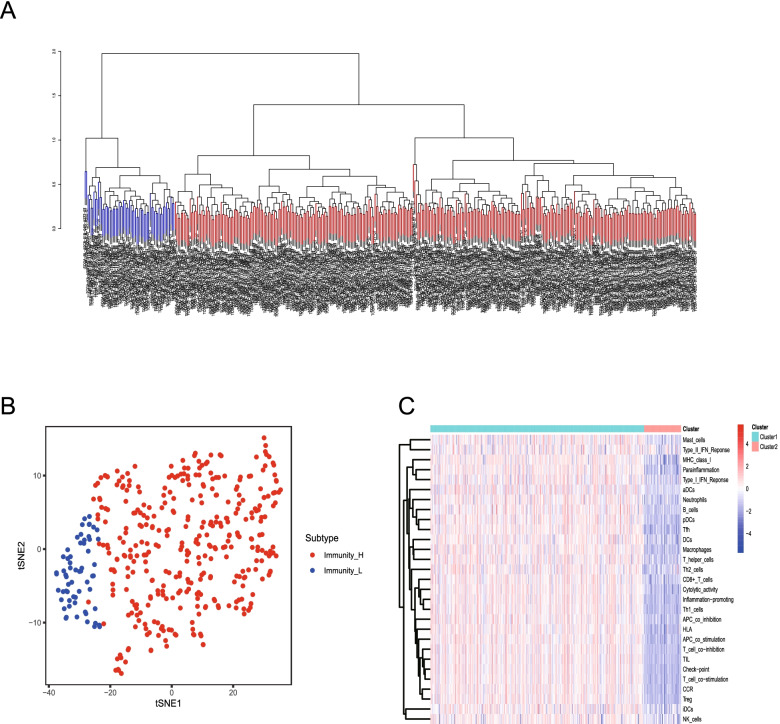
Identification of the immune gene subtypes of BLCA. **A.** Unsupervised clustering of BLCA samples in TCGA queues. **B.** Point diagrams of different immune gene subtypes identified using the t-SNE algorithm. **C.** Concentration values of 29 immune genes in different immune gene subtypes, which were identified using unsupervised clustering analysis

In this study, we used ssGSEA to quantify the concentration levels of 29 immune-related genes in BLCA. We identified 414 BLCA tumor samples as belonging to two immune gene subtypes (immunity_L and immunity_H) based on unsupervised clustering and on the ssGSEA scores (Fig. 1A). Figure 1B shows that the ssGSEA score can be used to distinguish immunity_L from immunity_H BLCA patients. The heat map shows the expression levels of 29 immune-related differential genes between the two different subgroups of immune genes (Fig. 1C).

### Correlation analysis of the BLCA immunogene subgroups, the tumor microenvironment, and the *HLA* gene

**Fig. 2:**
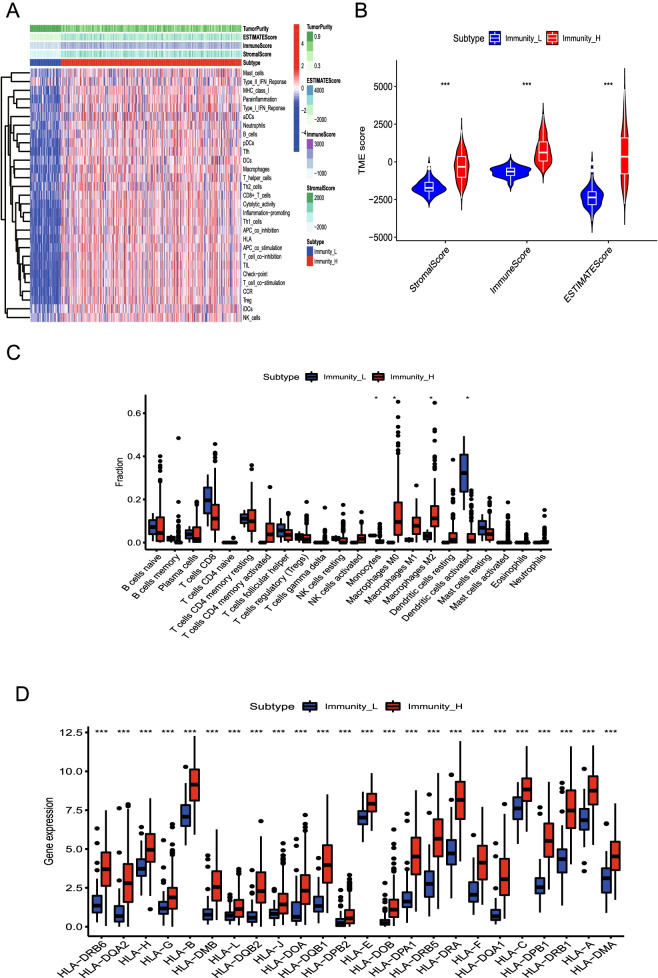
Correlation among the immune genome subtypes, the tumor microenvironment, and HLA genes. **A.** The immune score, matrix score, and tumor purity of the two BLCA immune gene subtypes were correlated with the levels of the 29 immune genes. **B.** Analysis of the differences in immune, stromal, and estimation scores between the two immune gene subtypes. **C.** The proportion of immune cells between the two immune gene subtypes is significantly different. **D.** Analysis of the differences in *HLA* gene expression between the two different immune gene subtypes

We applied the ESTIMATE algorithm to determine the ESTIMATE, stromal, and immune scores of the BLCA tumor microenvironment. Figure 2A shows the immunity scores of the immunity_H group, which had higher stromal and ESTIMATE scores than the immunity_L group. In contrast, a lower level of tumor purity was observed for the immunity_H than the immunity_L group. In addition, there were significant differences in the immune, stromal, and ESTIMATE scores of the two BCLA immune gene subtypes (*P* < 0.001; Fig. 2B). Furthermore, we systematically estimated the proportions of 22 tumor immune cells in BLCA using the CIBERSORT algorithm. Compared to in the immunity_H group, higher levels of activation of the monocytes and dendritic cells were observed in the immunity L group (*P* < 0.05; Fig. 2C). Conversely, higher levels of expression of the macrophages M0 and M2 were observed (*P* < 0.05) in the immunity H group than in the immunity L group.

We also analyzed differences in the expression of HLA genes between the two BLCA immune gene subgroups (Fig. 2D). The HLA gene expression level between the immunity_L and immunity_H groups was found to significantly differ, whereas the level of expression of HLA genes was found to be higher in the immunity_H group than in the immunity_L group (*P* < 0.001).

### Gene function enrichment analysis and identification of differentially expressed immune genes between the BLCA immune gene subtypes

**Fig. 3:**
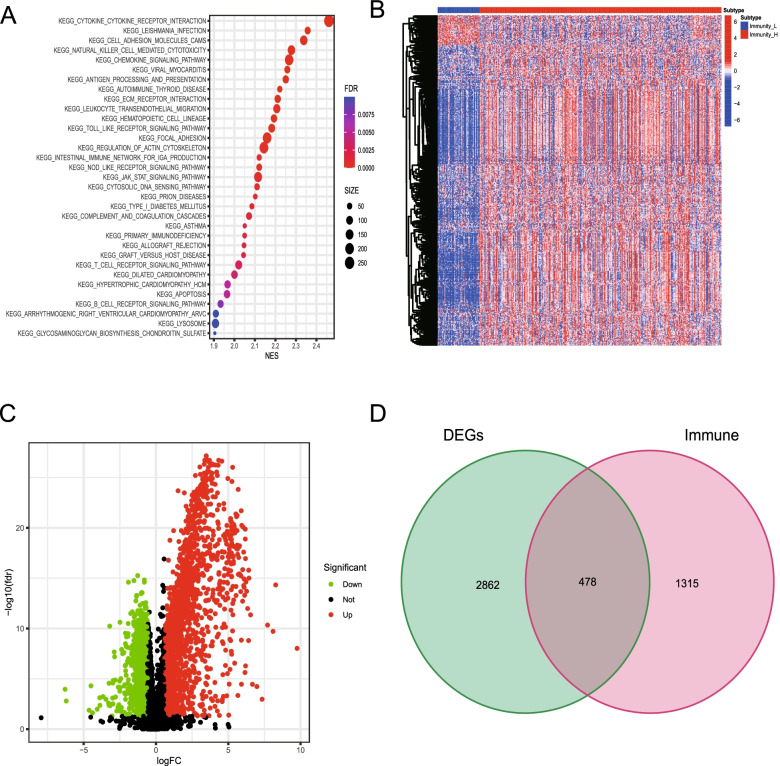
The functional annotation of the immune gene subtype was determined based on the differential expression of the immune genes. **A.** KEGG analysis results between the two different immune gene subtypes. **B.** Heat maps show the expression levels of the DEGs between the two immune subtypes and compare them between immunity_L and immunity_H. **C.** Expression of the DEGs in a volcanic map. Red indicates high expression, green indicates low expression, and black indicates medium expression. **D.** The Wayne graph shows the intersection of the DEGs with the set of immune genes, resulting in a total of 478 immune DEGs

To understand the biological pathway differences between the two different immune gene subtypes of BLCA, we performed GSEA using c2.cp.kegg.v7.symbols.gmt and c5.go.v7.symbols.gmt. The KEGG enrichment analysis results showed that cytokine receptor interactions, B-cell receptor signaling pathways, apoptosis, Toll-like receptor signaling pathways, and JAK STAT signaling pathways were significantly active in the immunity_H group (Fig. 3A). The results of GO enrichment analysis showed that in BP, pathways such as leukocyte interleukin 6 production, cell polarization, and positive regulation of cell adhesion were significantly active in the immunity_H group. In the case of MF, pathways such as cytokine binding, cytokine receptor binding, and extracellular matrix binding were significantly active in the immunity_H group. Regarding the CC, the extracellular matrix containing collagen, the membrane signal receptor complex, and the protein complex involved in cell adhesion were significantly related to the immunity_H group (Table 1).

**Table 1:** Gene function enrichment of the differentially expressed immune genes between the BLCA immune gene subtypes

NAME	*P* value	FDR
GOBP MONONUCLEAR CELL MIGRATION	< 0.001	< 0.001
GOBP POSITIVE REGULATION OF CYTOKINE PRODUCTION	< 0.001	< 0.001
GOBP INTERLEUKIN 6 PRODUCTION	< 0.001	< 0.001
GOBP LYMPHOCYTE MIGRATION	< 0.001	< 0.001
GOBP EXTERNAL ENCAPSULATING STRUCTURE ORGANIZATION	< 0.001	< 0.001
GOBP POSITIVE REGULATION OF CELL ADHESION	< 0.001	< 0.001
GOBP GRANULOCYTE CHEMOTAXIS	< 0.001	< 0.001
GOBP CELL CHEMOTAXIS	< 0.001	< 0.001
GOBP REGULATION OF IMMUNE EFFECTOR PROCESS	< 0.001	< 0.001
GOBP DEFENSE RESPONSE TO BACTERIUM	< 0.001	< 0.001
GOMF CYTOKINE BINDING	< 0.001	< 0.001
GOMF CYTOKINE ACTIVITY	< 0.001	< 0.001
GOMF CYTOKINE RECEPTOR BINDING	< 0.001	< 0.001
GOMF IMMUNE RECEPTOR ACTIVITY	< 0.001	< 0.001
GOMF CYTOKINE RECEPTOR ACTIVITY	< 0.001	< 0.001
GOMF INTEGRIN BINDING	< 0.001	< 0.001
GOMF EXTRACELLULAR MATRIX BINDING	< 0.001	< 0.001
GOMF EXTRACELLULAR MATRIX STRUCTURAL CONSTITUENT	< 0.001	< 0.001
GOMF COLLAGEN BINDING	< 0.001	< 0.001
GOMF ENDOPEPTIDASE REGULATOR ACTIVITY	< 0.001	< 0.001
GOCC EXTERNAL SIDE OF PLASMA MEMBRANE	< 0.001	< 0.001
GOCC COLLAGEN CONTAINING EXTRACELLULAR MATRIX	< 0.001	< 0.001
GOCC CELL SUBSTRATE JUNCTION	< 0.001	< 0.001
GOCC VACUOLAR LUMEN	< 0.001	< 0.001
GOCC ENDOCYTIC VESICLE MEMBRANE	< 0.001	< 0.001
GOCC FICOLIN 1 RICH GRANULE MEMBRANE	< 0.001	< 0.001
GOCC PROTEIN COMPLEX INVOLVED IN CELL ADHESION	< 0.001	< 0.001
GOCC VESICLE LUMEN	< 0.001	< 0.001
GOCC PLASMA MEMBRANE SIGNALING RECEPTOR COMPLEX	< 0.001	< 0.001
GOCC LYSOSOMAL LUMEN	< 0.001	< 0.001

*FDR* False discovery rate

To screen for the DEGs associated with immunity in the two BLCA immunogen subtypes, we first identified gene expression differences between the immunity_L and immunity_H groups. 3,340 DEGs were identified in total. The expression levels of these genes are presented in a heat map (Fig. 3B), while a volcanic map shows the differences in the expression of these genes between the two immune gene subtypes (Fig. 3C). Next, we used the previously obtained differences to express the intersection of genes and immune genes. Finally, 478 BLCA immune-related DEGs were obtained (Fig. 3D).

### Construction of the immunity gene signature related to BLCA

**Fig. 4:**
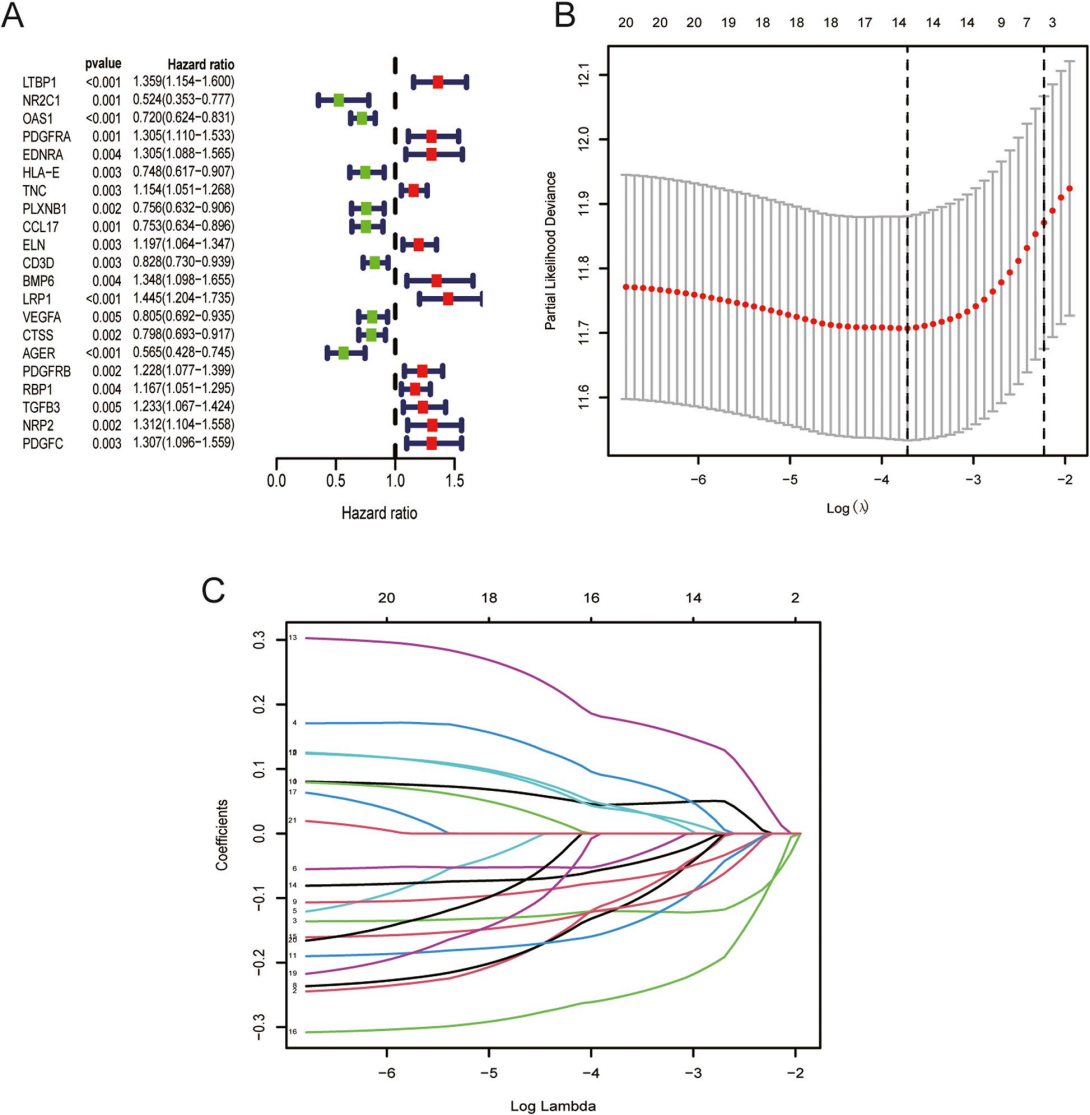
Construction of the BLCA immune-related gene signature. **A.** Single-factor Cox analysis identified 21 immune genes associated with the prognosis of patients with BLCA. **B**-**C.** LASSO regression identified four of the most predictive immune genes in TCGA queue

We performed Univariate Cox analysis of the immune genes based on their differential expression to identify the immune genes associated with BLCA prognosis, using *P* < 0.01 as the threshold. A total of 21 immune genes associated with the prognosis of patients with BLCA were obtained (Fig. 4A). The LASSO regression model was used to screen the BLCA signature genes for the development of a new BLCA gene signature (Fig. 4B, C). Finally, four BLCA signature genes were used to develop the gene signature. The formula used for developing the BLCA gene signature is as follows: risk score = 0.1601 × expression of *RBP1* + (-0.2471) × expression of *OAS1* + (0.2570) × expression of *LRP1* + (-0.4403) × expression of *AGER*.

**Fig. 5:**
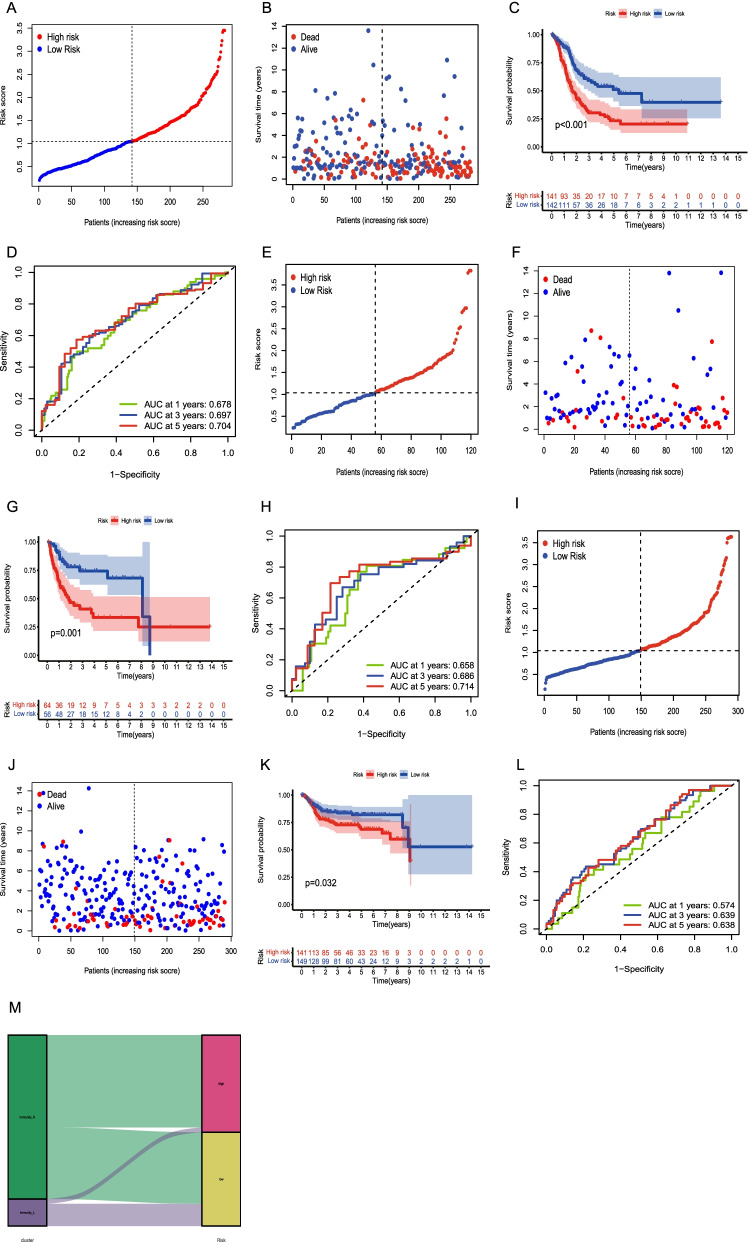
Functional evaluation and validation of the gene signature. **A**, **E**, **I**. Patient distribution based on the risk score. **B**, **F**, **J**. Distribution of the patient survival times based on the risk score. **C**, **G**, **K** Survival analysis between the high and low-risk groups. **D**, **H**, **L**. The ROC curves verified the prognostic value of the gene signature. **M**. The alluvial map shows that the immunity_L group was more related to the low-risk group

The TCGA cohort was randomly segregated into test and training groups. Based on the BLCA gene signature, the patients with BLCA in the training group were further segregated into low-risk and high-risk groups (Fig. 5A). An increase in the risk score was associated with a decrease in the survival time of the BLCA patients (Fig. 5B). The overall survival of the patients in the low-risk group was observed to be longer than that of patients in the high-risk group, based on the Kaplan–Meier survival curve results (*P* < 0.001, Fig. 5C). To evaluate the predictive power of the BLCA gene signature, the total survival projections for the 1-, 3-, and 5-year periods based on the gene signature were 0.678, 0.697, and 0.704, respectively (Fig. 5D). This indicated that the BLCA gene signature has good predictive power. In the patients with BLCA, risk score, TNM stage, stage, and age were associated with poor survival in the Univariate analysis results. The potential use of the risk score of the gene signature as independent prognostic factor is illustrated by the multivariate analysis results (HR: 1.76; 95% CI: 1.43–2.16; *P* < 0.001; Tables 2 and 3).

**Table 2:** Univariate analysis results show that the risk scores are associated with a shorter OS, age, stage, and TN stage

	HR	HR.95L	HR.95H	*p*value
Age	1.034418	1.017015	1.05212	*P* < 0.001
Gender	0.874448	0.615043	1.243263	0.454922
Grade	4.403557	0.61378	31.59327	0.140357
Stage	1.837732	1.466205	2.303401	*P* < 0.001
T	1.643298	1.289043	2.094909	*P* < 0.001
N	1.55842	1.319098	1.841162	*P* < 0.001
riskScore	1.853321	1.53359	2.23971	*P* < 0.001

**Table 3:** Multivariate analysis shows that the risk score of the gene signature can be used as an independent prognostic factor

	HR	HR.95L	HR.95H	*p*value
Age	1.031691	1.014011	1.04968	*P* < 0.001
Stage	1.193283	0.770596	1.847822	0.428359
T	1.20793	0.887669	1.643737	0.229414
N	1.30408	0.963671	1.764734	0.085395
riskScore	1.757823	1.430016	2.160774	*P* < 0.001

*T* Tumor, *N* Lymph nodes

### Functional verification of the BLCA-related gene signature

To verify the reliability of the application of the BLCA gene signature to different populations, we calculated the risk scores of patients with BLCA in the test group and GEO queue using the same formula. The test group and GEO queue were used to verify that the results of the queue are consistent with the previous results. Depending on the risk scores of the gene signature, the patients with BLCA in the validation queue were segregated into low-risk and high-risk categories (Fig. 5E, I). An increase in the risk score was associated with lower survival outcomes in patients (Fig. 5F, J). Moreover, compared with the survival results of the high-risk group, the results of the low-risk group were superior, as seen from the results of the Kaplan–Meier survival curve (test group: *P* = 0.001; GEO queue: *P* = 0.032; Fig. 5G, K). In the test group, the total survival projections for one, three, and five years based on the AUC results were 0.658, 0.686, and 0.714, respectively (Fig. 5H). In the GEO cohort, the total survival projections for one, three, and five years based on the AUC results were 0.574, 0.639, and 0.638, respectively (Fig. 5L). The potential to accurately predict the prognosis of the patients with BLCA and the applicability of the gene signature in different populations was confirmed by these results. The relationship between the risk score and immunophenotype was analyzed and visualized using an alluvial map. Lower risk scores were observed in the immunity L group(Fig. 5M).

### Correlation between gene signature and immunophenotyping, immune cells, and somatic mutation

**Fig. 6:**
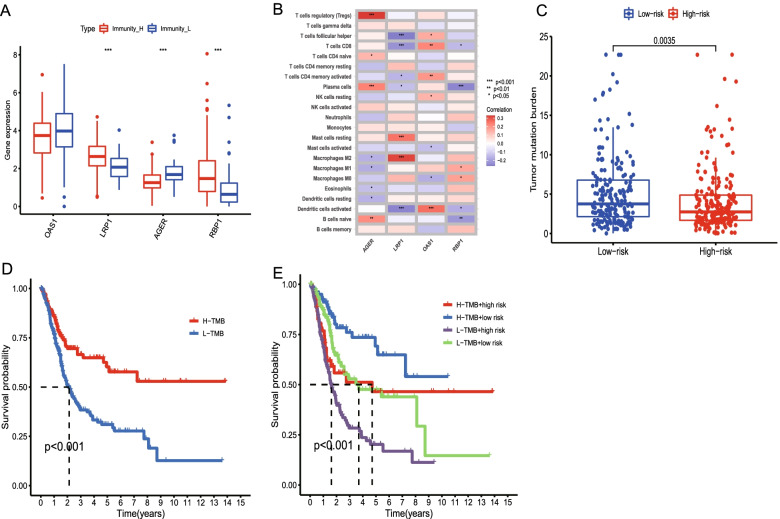
Correlation between gene signature and immunophenotyping, immune cell and somatic mutation. **A.** The multiple box diagram shows the expression differences of four characteristic genes between the immunity_L and immunity_H groups. **B.** Heat maps show the correlation between the four characteristic genes that build the gene signature and the immune cells. Blue indicates a negative correlation; red indicates a positive correlation. **C.** Tumor mutation load (TMB) between the two different immune gene subtypes. **D.** The Kaplan–Meier curve was used to analyze the survival outcomes of the BLCA patients between the high and low TMB groups. **E.** The Kaplan–Meier curve was used to analyze the risk scores and survival of patients in the TMB group. In both the high and low TMB groups, the patients with BLCA in the low-risk group have survival advantages

Compared to that in the immunity_L group, the expression of RBP1 and LRP1 in the immunity_H group was higher than that in the immunity_L group. Conversely, the expression of AGER in the immunity H group was lower than that in the immunity L group (Fig. 6A). Correlation between the four characteristic genes and the immune cells significant for the development of the BLCA. The results showed that AGER was significantly positively correlated with regulatory T cells, plasma cells, and B cells. LRP1 was significantly positively correlated with resting mast cells and macrophages M2, while it was significantly negatively correlated with follicular helper T cells, dendritic cells, and CD8T cells. OAS1 was significantly positively correlated with CD8T cells, memory CD4T cells, and dendritic cells, whereas RBP1 was significantly negatively correlated with plasma and B cells (Fig. 6B).

Studies have shown a correlation between mutations in somatic cells in tumor tissues and immune therapy response. We analyzed the distribution patterns of the tumor mutation load (TMB) in the low- and high-risk groups. The frequency of mutations in the low-risk group was higher than that in the high-risk group (Fig. 6C). Compared with the patients with a low mutation frequency, the survival results of the patients with a high mutation frequency were found to be markedly better, as revealed by the survival analysis results (Fig. 6D). Moreover, in both the low and high mutation groups, the patients in the low-risk group indicated a clear advantage of survival (Fig. 6E).

### Predictive value of the gene signature for immunotherapy effects

**Fig. 7:**
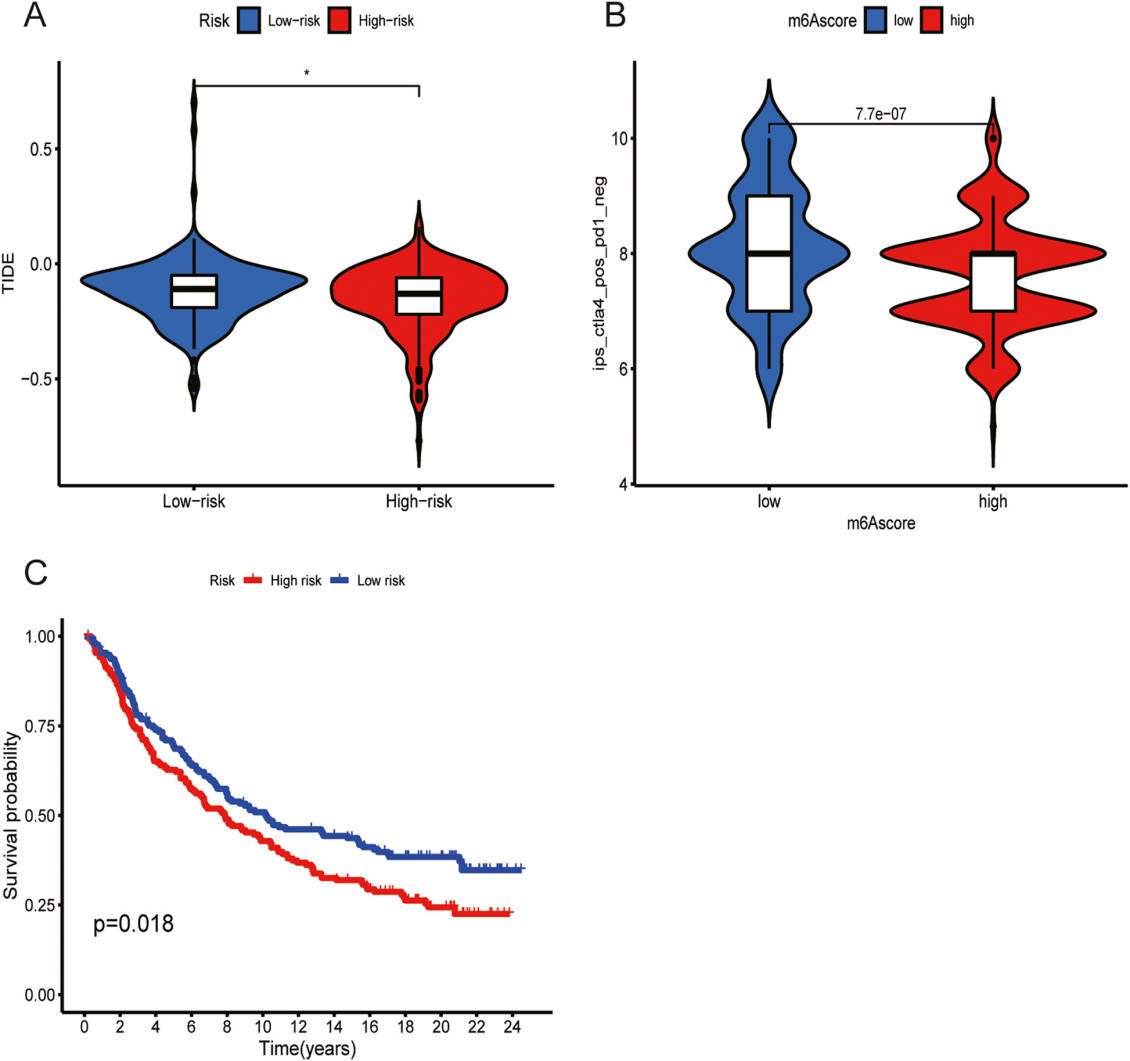
Prediction of the effects of immunotherapy using the gene signature. **A.** TIDE distribution differences between the high- and low-risk groups. **B.** Assessment of the differences in the effectiveness of CTLA4 treatment between the high- and low-risk groups. **C.** Kaplan Meier analysis shows that patients in the low-risk group were associated with better survival outcomes compared with patients in the high-risk group

An analysis of the expression of the Tumor Immune Dysfunction and Exclusion (TIDE) in the high-risk and the low-risk groups revealed higher incidence of TIDE in the low-risk group than in the high-risk group (*P* < 0.05; Fig. 7A). In the CTLA4 treatment group, patients in the low-risk group exhibited better outcomes (*P* = 7.7e − 07; Fig. 7B). More importantly, we used the imvigor210 dataset to evaluate the ability of our model to predict the effect of immunotherapy based on four characteristic genes. Compared to the patients in the high-risk group, the patients in the low-risk group were associated with better survival outcomes in the Kaplan Meier analysis (Fig. 7C).

### Nomogram prediction model construction and evaluation

**Fig. 8:**
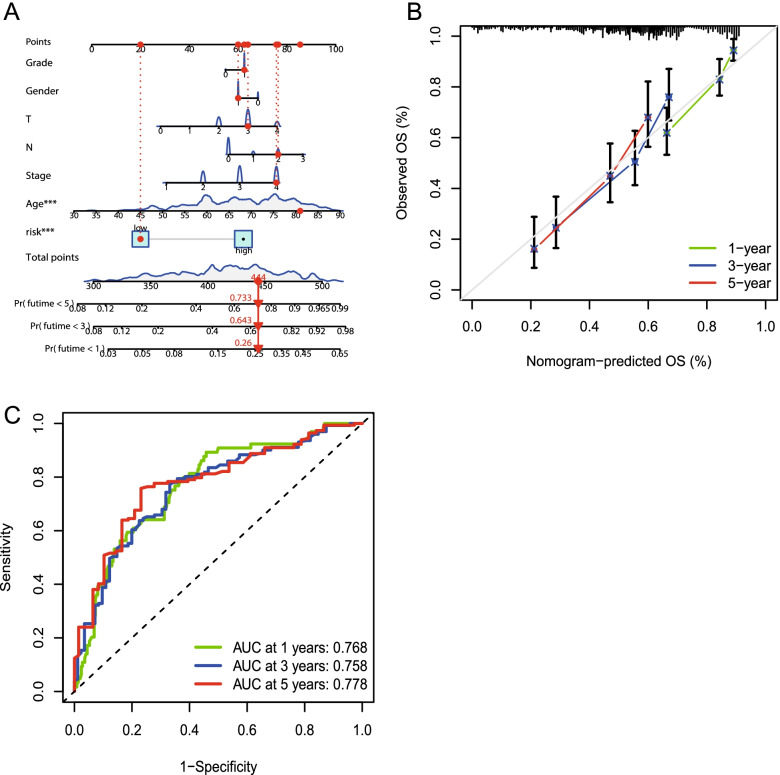
Evaluation and validation of the predictive power of the column charts. **A.** Line charts based on the risk scores and age, gender, grade, stage, and TN staging. **B.** Line charts predict the prognostic time-dependent ROC curves at 1, 3, and 5 year time points. **C.** Calibration diagram of the 1-, 3-, and 5-year lifetime line charts

We built a line chart based on clinical characteristics, such as the BLCA-related genes, age, sex, and stage, to predict the total survival rates of patients with BLCA over one, three, and five years (Fig. 8A). The total survival projections for one, three, and five years based on time-dependent ROCs were 0.768, 0.758, and 0.778, respectively (Fig. 8B). We also used calibration charts to evaluate the predictive effect of this line chart on the total lifetime of patients with BLCA (Fig. 8C). These results suggest that the genes that combine clinical characteristics have potential clinical benefits.

### Functional enrichment analysis between high- and low-risk groups

**Fig. 9:**
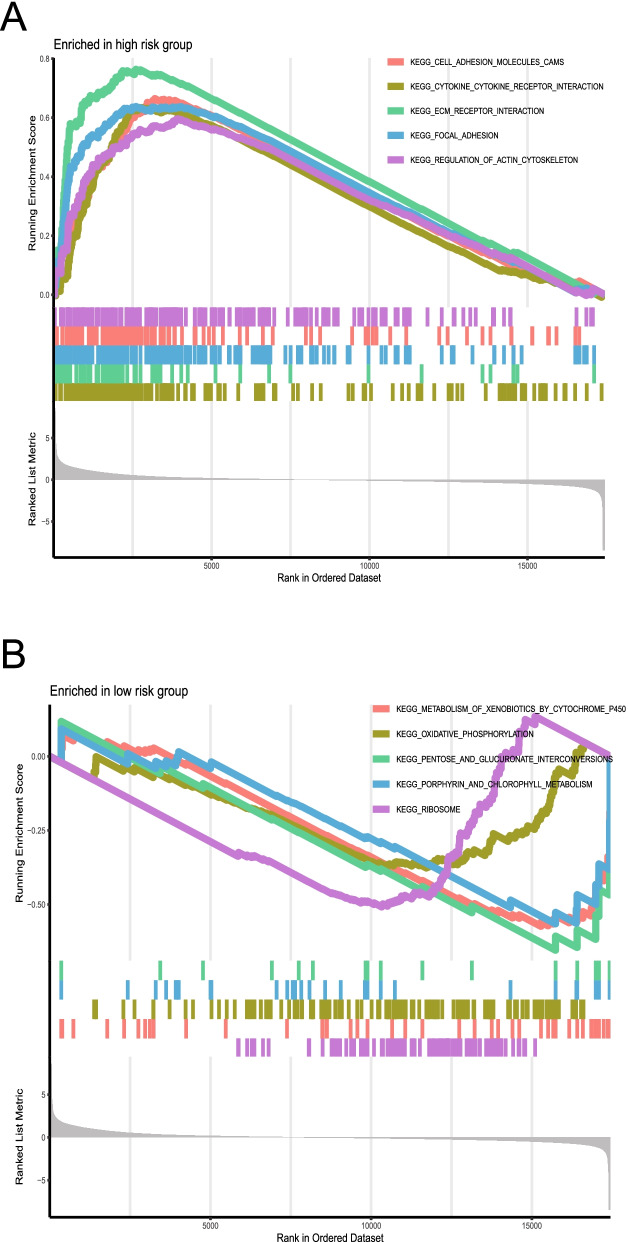
Functional enrichment analysis shows different biological pathways between high- and low-risk groups. **A** The high-risk group was mainly enriched in cell adhesion molecules, cytokine receptor interaction, ecm receptor interaction, focal adhesion, and regulation of actin cytoskeleton. **B** The low-risk group was mainly enriched in the metabolism of cytochrome P450 to heterologous substances, oxidative phosphorylation, mutual transformation of pentose and glucuronic acid, porphyrin and chlorophyll metabolism, and ribosome

The GSEA results revealed differences in biological pathways between the high- and low-risk groups. The high-risk group was mainly enriched in cell adhesion molecules, cytokine receptor interaction, ecm receptor interaction, focal adhesion, and regulation of actin cytoskeleton (Fig. 9A). Conversely, the low-risk group was mainly enriched in metabolism of cytochrome P450 to heterologous substances, oxidative phosphorylation, mutual transformation of pentose and glucuronic acid, porphyrin and chlorophyll metabolism, and ribosome (Fig. 9B).

## Discussion

BLCA is a common malignant genitourinary tract tumor [39]. However, progress in bladder cancer treatment has been limited. Although surgery is still the preferred treatment for BLCA, patients have a high rate of postoperative recurrence [40]. Hence, new strategies for treatment need to be developed in which the identification of BLCA biomarkers is imperative. In the present study, the relative abundance of the 29 immune genes in the BLCA samples was calculated using ssGSEA. Two different BLCA immune gene subtypes (immunity_L and immunity_H) were determined using unsupervised clustering methods based on ssGSEA scores. We screened differentially expressed immune genes between the two immune gene subtypes and identified the immune genes associated with prognosis using univariate cox analysis. Finally, we built a gene signature related with BLCA based on the immune genes associated with prognosis. The BLCA-related gene signature could divide patients into low- and high-risk groups, and the patients in the low-risk group had significant survival advantages and showed significant immunotherapy effects. In summary, we aimed to provide a new gene signature to promote the personalized treatment of BLCA.

We observed that the expression levels of monocytes and dendritic cells activated in the immunity_L group were higher than those in the immunity_H group, whereas the expression levels of macrophages M0 and M2 in the immunity_L group were lower than those in the immunity_H group. Besides being mononuclear phagocytes, the monocytes were found to be significant regulators of cancer development and progression. Contrasting roles were played by the different subsets in the prevention of cancer cell metastasis and diffusion, along with the promotion of tumor growth [41, 42]. Recent research by Wang et al. revealed that intermediate monocytes can be induced by IFN-γ to inhibit cancer metastasis by promoting natural killer cell activation by FOXO1 and interleukin 27 [43]. Significant roles are played by the macrophages in inflammation and innate immunity [44]. M2 macrophages are a subtype of macrophages that can inhibit inflammatory response and promote angiogenesis and tissue remodeling and repair [45]. Previous studies have shown that bladder cancer cells can stimulate BMP4 to induce macrophages and polarize the M2 phenotype, thereby promoting bladder cancer development [46]. The levels of RBP1 and LRP1 were significantly higher in the immunity H group than in the immunity_L group.

We analyzed the biological pathway differences between the two immune gene subgroups of BLCA using GSEA. Cytokine receptor interactions, receptor signaling pathways, JAK STAT signaling pathways, leukocyte interleukin 6 generation, and mass membrane signal receptor complexes were more active in the Immunity_H group. Having a crucial role in the immune system, Toll-like Receptors (TLRs) are members of the pattern recognition receptor family. TLRs can mediate the identification of the pathogen-related molecular patterns derived from pathogens and damage-related molecular patterns from damaged cells, further driving host innate and adaptive immunity activation [47–49]. Chronic inflammation can promote tumor development. TLR is an effective activator of the inflammatory response [50]. TLR expression in cancer cells can trigger an NF-B signaling cascade, the production of inflammatory cytokines, and anti-apoptosis proteins, thereby promoting tumor growth [51]. In several cancers, TLR4 has been indicated to play a significant role. TLR4 activation increases the expression of VEGF and TGF-β1 in prostate cancer cells, which promotes tumor development [52]. Overexpression of TLR4 has been associated with poor prognosis in breast and colon cancers [53, 54]. In addition, TLR4 has been found to have antitumor activity in skin cancer [55]. Previous studies have found that TLR4 is less expressed in non-myo-inundated bladder cancer [56]. TLR4 may have a significant impact on BLCA development. The JAK/STAT signaling pathway is an important component of functional reactions. Three major proteins, a cell surface receptor, JAK, and STAT, are involved in JAK/STAT signaling [57]. An abnormal activation of the JAK/STAT signaling pathway may be a key factor promoting tumor development [58]. STAT3 promotes cell cycle processes by promoting the activation of cell cycle protein-dependent kinases. STAT5 protects cells from apoptosis by activating the transcription of Bcl-x to produce the anti-apoptosis protein Bcl-XL. The inhibition of STAT activity reduces cell proliferation and increases apoptosis [59]. Therefore, antagonist JAK/STAT signaling may help to inhibit the development of BLCA.

In the present study, we constructed a gene signature containing four BLCA signature genes (*RBP1*, *OAS1*, *LRP1*, and *AGER*). Retinol-binding protein type 1 (RBP1) is a cytosolic carrier responsible for regulating retinol homeostasis in human tissues [60, 61]. Previous studies have observed abnormal expression of RBP1 in a variety of human cancers [62–66]. RBP1 is responsible for regulating intracellular retinoic acid homeostasis, which is related to morphogenesis, cell proliferation, and differentiation. Therefore, the deletion of RBP1 may lead to tumorigenesis in bladder cancer. The abnormal CpG hypermethylation of the RBP1 gene promoter may be related to bladder cancer development [66]. DNA damage is an important step in cancer development [67]. Poly (ADP-Ribose) is a quick synthesis at the DNA damage site that facilitates repair. The high expression of OAS1 promotes the ability of cancer cells to survive DNA damage by reducing PAR synthesis, thus preventing cell death [68]. Low-density lipoprotein receptor-related protein (LRP) is a transmembrane receptor that plays a role in cell swallowing, cell signaling, and other cell protein transport [69]. LRP1 can act as a receptor for medium-term growth factors, signaling through ERK1/2 to induce the expression of internalized metalloproteinase, and promote the survival of micro-metastasis, thereby promoting cancer progression [70–73]. AGER is a member of the superfamily of cell surface receptors for immunoglobulins. A study by Wang et al. has shown that AGER overexpression, which is associated with age, reduces the proliferation, invasion, and migration of lung cancer cell lines and increases apoptosis [74]. However, the mechanisms underlying the actions of these characteristic genes in BLCA require further studies.

The survival of patients in the TCGA-BLCA queue was measured to determine the prognostic value of the gene signature. The gene signature could divide the BLCA patients into high- and low-risk groups based on the risk scores, and patients in the low-risk group had significant survival advantages. The immunity_L group was more associated with the low-risk group. In addition, we monitored the progress of BLCA through the risk curve generated based on the risk score. The ROC curve showed that the gene signature has good predictive ability. All results were validated in the test group and GEO queue. This suggests that our gene signature can be applied to other individuals in queues. The risk score, being an independent predictor of the total survival of the BLCA patients, was confirmed further by multivariate analysis. Moreover, our genetic signature risk scores were significantly associated with immune response predictors, such as immune scores, and TIDE. Good response to immunotherapy was displayed by the low-risk group patients. This gene signature facilitates the personalized treatment for bladder cancer. As a result, our gene signature is expected to be a valuable tool for evaluating the prognosis of patients with BLCA.

The correlation between the genes that build immune cells and the gene signature was also analyzed. The four characteristic genes were significantly associated with immune cells, such as regulatory T cells, plasma cells, B cells, resting mast cells, macrophages M2, follicle-assisted T cells, and dendritic cells. Previous studies have shown that tumor-related macrophages can promote and may even be necessary for angiogenesis in bladder cancer [75, 76]. Macrophages M2 are phenotype phenocytes and may promote BLCA development by inducing immunosuppression, angiogenesis, and cell invasion [77]. Dendritic cells are considered the most effective anti-cancer immunosuppressors [78]. Previous studies have found the presence of immature and minimally activated dendritic cells in the urine and bladder of BLCA patients [79]. We believe that the reduction or inhibition of dendritic cells in the tumor microenvironment may be a key factor promoting BLCA development. Regulatory T cells (Tregs) play an important role in cancer immune escape [80]. Poor prognosis in a variety of tumor patients was revealed by the high levels of infusion of Treg in the associated tumor tissues [81]. We believe that the removal of Treg cells from the BLCA tumor microenvironment has the potential to stimulate and enhance antitumor immune response in patients with BLCA.

The present study had some limitations. First, we only analyzed data from public databases. Second, we need to examine larger queues of BLCA patients receiving immunotherapy in the future to verify our results.

## Conclusions

In the present study, we developed and validated a gene signature containing four genes. The gene signature could be used as an independent prognostic factor for BLCA patients. The gene signature may help monitor the occurrence and prognosis of BLCA. Moreover, the clinical response of patients with BLCA to immunotherapy can be predicted using the gene signature. Our findings thus provide new insights into personalized BLCA immunotherapy.

## Data Availability

The BLCA data used for analysis in this study were obtained from the TCGA database (https://tcga-data.nci.nih.gov/tcga/) and the GEO database (http://www.ncbi.nlm.nih.gov/geo/).
